# From welfare states to welfare sectors: Explaining sectoral differences in occupational pensions with economic and political power of employees

**DOI:** 10.1177/0958928715611006

**Published:** 2015-12

**Authors:** Tobias Wiß

**Affiliations:** Johannes Kepler University Linz, Austria

**Keywords:** Occupational pensions, power resources, skills, social policy, trade unions, welfare state

## Abstract

Studies analysing welfare have previously focused on countries as units. In the course of pension cuts and the increasing importance of occupational welfare, our traditional understanding of a homogeneous welfare state is being challenged. In this article, I distinguish between both economic individual power (employee skills) and political collective power (trade unions), and their relation with different occupational pensions. A combined analysis by both factors is not common, where employee skills and power resources are traditionally treated as separate, rival explanations of public welfare. Combining the ‘method of difference’ with the ‘method of agreement’, the article first presents the within-country variety of occupational pensions in Germany, Italy, the United Kingdom and Denmark. Occupational pensions in the same economic sectors across countries are then used as the units of analysis in order to illustrate the plausible determinants of economic individual power and political collective power.

Many European countries have seen a retrenchment in public social policies over recent decades due to fiscal restrictions and demographic changes. Welfare states are in flux and non-state social policy is promoted in a large number of countries in order to cope with these challenges. In changing welfare states, occupational welfare represents a very important potential compensation for retrenchment (Trampusch, 2009). Although the comparative welfare state literature has improved our understanding of retrenchment processes (Obinger et al., 2010; Schludi, 2005; Seeleib-Kaiser, 2008), these studies have focused on public pensions and countries as units of analysis, neglecting within-country and sector differences.

International research generally refers to the main public and occupational pension scheme for country comparisons (Greve, 2007; Immergut et al., 2007; Trampusch, 2009; for more within-country analyses, see Ebbinghaus, 2011b; Meyer et al., 2007). The same holds true for studies on the conditions for occupational family policy. For example, Seeleib-Kaiser and Fleckenstein (2009) rely on the views of managers in stock-listed companies. Taking only one pension scheme or the average representing the country leads to misinterpretations. Occupational pension schemes – as occupational welfare in general – differ across economic sectors, resulting in different levels of coverage (for a cruder analysis, see Seeleib-Kaiser et al., 2012), contributions and benefits for employees. These differences in social policy at the sectoral level challenge the understanding of homogeneous welfare states and are essential for country and regime classifications. Mapping variety first within countries for – at least some – different sectors and, second, for different sectors across countries provides a more realistic picture of pension systems and calls for more accuracy of country comparisons and the projection of future old-age incomes (for the last point, see Peeters et al., 2014).

As we will see below, cross-sectoral differences are similar in three of the four studied countries. Only Denmark is equipped with more homogeneous occupational pension schemes. Nevertheless, what do the patterns of cross-sectoral differences look like in these countries and why is Denmark different? To find an answer, the present article develops a framework distinguishing between employees’ economic and individual power (employee skills) and political and collective power (trade unions). While the former alone is associated with generous occupational pension schemes for employees with high-general or high-specific skills, the intervention of the latter gives power to economically weak employees, resulting in high coverage rates. Although developed for country comparisons of market economies and public welfare, for the first time their explanatory power is considered in a joint perspective and applied to sectoral comparisons of occupational welfare. From an empirical point of view, the article systematically compares countries and sectors using national data not only on coverage rates but also on contribution rates. Skills are measured across countries and sectors based on data from the European Labour Force Survey (LFS).

In the section ‘Employees’ economic and political power resources’, I explain the theoretical framework by critically referring to skills and power resources. Then I point to the overall pension system and the scope for occupational pensions. Afterwards, applying the ‘method of difference’, I focus on different occupational pension schemes across sectors within each country using national resources in order to map their variety. In terms of economic sectors, the article mainly refers to public administration, finance and insurance, manufacturing, construction, hospitality and administrative and support services. Finally, the article provides evidence for the association of economic power and/or political power with widespread and generous occupational pension schemes in different economic sectors across countries (‘method of agreement’).

## Employees’ economic and political power resources

Skill-related approaches (economic power) and power resources approaches (political power) are powerful explanators for the differences in public social policies. Instead of treating power resources and skills as rival explanations, in this section, I argue that they complement each other, where both grant different employees’ power. In general, state regulations influence the scope of occupational pensions as well as the preferences of employers and trade unions (Shalev, 1996; Trampusch, 2013). Since public pension schemes are the same in all the economic sectors of a country, they cannot be utilized as an explanation for sectoral differences. Instead, the skills of employees and power resources of trade unions differ between economic sectors.

### Economic and individual power of employees

Employer-centred approaches argue employers have a genuine interest in public social policy (Hall and Soskice, 2001; Mares, 2003a), and that the incidence of employment-related risks such as accidents and sickness, firm size and employee skills are decisive for the support of different welfare systems (Mares, 2001, 2003b). The main risk employers are faced with in the case of pensions is longevity. Employees with high life expectancy receive pensions for a long period, financed by the employer and/or employee contributions. Theoretically, high-risk industries should be more interested in redistributive and public social policies (Mares, 2001: 194) and less in non-redistributive private occupational pensions. However, even in high-risk industries where employees have a long average life span such as finance and public administration, employers offer widespread and generous occupational pensions. The longer employees live, the longer is the payout period and the more financial resources have to be accumulated over the working life (in the case of employer-financed occupational pensions). Industry-wide or firm-based occupational pensions either lack inter-occupational redistribution or have only a very narrow risk pool (in contrast to a nationwide scheme), allowing for a broader risk redistribution.

Next to risk, firm size has been a common explanatory factor for occupational welfare (Mares, 2001; Trampusch and Eichenberger, 2012). Generally, owing to economies of scale, large firms with human resource departments able to organize firm-based welfare are more active in occupational pensions than small- and medium-sized firms (Bridgen and Meyer, 2005: 772). However, size alone is not necessarily decisive. In her theoretical model, Mares (2001: 201) treats firm size and skill intensity as one dimension. Therefore, we could assume that large firms with less qualified employees have a lower interest in occupational welfare than large firms with highly qualified employees have (for the example of firm-level vocational training, see Mares, 2003b: 244). Furthermore, strong unions may extend collective agreements industry-wide, including small firms. Therefore, for this analysis, the size argument is only of a peripheral interest.

Certain welfare benefits enable employees to invest in risky (specific and non-portable) skills, which is of interest for their employers. In order to minimize the risk of unemployment for these specifically qualified employees, employers sometimes support social policy such as unemployment insurance and labour market protection measures (Estevez-Abe et al., 2001; Iversen, 2005). Hence, different skill requirements of employers shape their social policy preferences and different skills are also responsible for different social policy preferences of employees (Iversen and Soskice, 2001). In addition, next to preferences, skills lead to different power resources of employees. Therefore, I refer to skills as economic and individual power, where certain skills materialize in employee power vis-à-vis the employer, who is in need of certain skills. Employees with high-general or specific skills, due to their high or specific human capital, are in positions in which they can make use of their economic power to ask for additional benefits such as occupational pensions. At the same time, highly and specifically qualified employees are of high value for firms relying on their expert knowledge and innovations, especially in times of labour shortages. Through the provision of occupational welfare, employers can attract employees, bind them to the firm and motivate their investments in specific skills. In line with Korpi (2006), the economic power of employees makes employers consenters and not protagonists of (occupational) welfare.^1^ Nevertheless, skills alone are not sufficient to explain sectoral differences in countries such as Denmark, where occupational coverage rates are very high even in economic sectors with low-qualified employees (e.g. in the hospitality sector).

### Political and collective power of employees

A second explanation, political collective power, represents an alternative path to widespread and generous occupational pensions. According to power resources theories, the emergence and shape of welfare states are related to the power and preferences of their actors (Korpi, 1983).

In general, the stronger trade unions within a country are, the higher the level of public welfare. By contrast, in countries with very weak trade unions, we tend to expect lower public benefits. For employers, a high degree of decommodification and high welfare expenses mean higher social contributions or tax payments, lower work incentives for employees and less control over employees (see Paster, 2011: 5). Additionally, due to high levels of control over occupational welfare, employers prefer firm-based welfare compared with public social policy set by politicians (Mares, 2001). Nevertheless, in some political situations (e.g. in the case of strong unions), employers agree to political and power constraints on the voluntary introduction or expansion of social policies (Korpi, 2006; Paster, 2011, 2013). If no agreements are made, they have to fear negative developments in occupational pensions such as mandatory occupational pension schemes for all employees with obligatory employer contributions. With regard to occupational welfare, depending on the political context and power of trade unions, employers may offer higher benefits to a wider circle of employees, avoiding the more binding elements and state intervention. For trade unions, generous public benefits are the preferred choice, but occupational welfare can be an alternative strategic choice in times of retrenchment and shrinking power resources. Trade unions accept reforms with cutbacks as long as they remain veto players or even gain responsibilities and future veto power in new fields such as occupational pensions via collective agreements (Lindvall, 2010; Wiß, 2012b).

At the sectoral level, strong trade unions and high collective bargaining rates are supposed to favour generous occupational welfare benefits based on collective agreements (for the country level, see Hacker, 2002; Trampusch, 2009). Trade unions with strong power resources are more likely to be successful in negotiations with employers than weak trade unions. However, power resources – as skills – alone are not sufficient to explain sectoral differences, since occupational pension coverage is, for example, very high in finance and insurance (see, for example, Seeleib-Kaiser et al., 2012) despite weak trade unions (Visser, 2013).

### A joint perspective

When considering employer-centred approaches and power resources approaches, both strands have complementary elements eliminating some shortcomings. Skills alone are not able to solve the empirical puzzle of widespread occupational pension schemes in sectors with low-qualified employees (e.g. the hospitality sector in Denmark). Furthermore, political power resources do not explain the generous occupational pension schemes in sectors with weak trade unions (e.g. finance and insurance). Employer-centred approaches mainly refer to skills and do not explicitly link them with employees’ power, while traditional power resources approaches only focus on political or collective power without considering individual power. By offering a joint perspective, these two paths lead to widespread and generous occupational pensions. In the first, employees with high-general or specific skills make use of their economic power in individual negotiations to demand occupational pension plans with (high) employer contributions. In the second, trade unions intervene and make use of their political and collective power in order to negotiate occupational pension schemes for economically weak employees, who – due to their low-general skills – lack individual economic power. By contrast, the lack of both employees with high or specific skills and strong trade unions results in scattered and rudimentary occupational pension schemes with low or no employer contributions.

## In search of evidence: similar sectors in different countries

The combination of the ‘method of difference’ with the ‘method of agreement’ (Mill, 1882) helps to control for other factors causing the outcome. I first apply the ‘method of difference’ for the comparison of occupational pensions in different sectors within each country in order to identify similar patterns in all countries. The ‘method of agreement’ will then demonstrate whether similar associations of economic and political power with occupational pensions exist for six economic sectors in different countries. I do not apply quantitative or set-theoretic methods due to the small number of cases and because the aim is not to test a large number of hypotheses. Rather, the aim is to offer broad comparative evidence for the role of employees’ economic and political power in occupational pensions.

In order to cover different institutional characteristics, I select countries with a range of welfare regimes, production regimes and systems of industrial relations. Germany represents conservative welfare regimes and continental coordinated market economies with cooperative industrial relations. Italy is typical of Mediterranean welfare regimes and mixed market economies with polarized and fragmented industrial relations. The United Kingdom is an illustration of a liberal welfare regime, with liberal market economies and pluralist and fragmented industrial relations. Finally, Denmark is a prime example of a Nordic welfare regime, with a social democratic coordinated production regime with corporatist industrial relations.^2^

For the selection of sectors, I refer to employee skills and trade union power. Various sectors require different employee qualifications. For the measurement of skills, I use the International Standard Classification of Occupations (ISCO)-08 classification and data from the EUROSTAT LFS, which allow us to measure the occupational characteristics in different sectors across countries (see Estevez-Abe et al., 2001; Fleckenstein et al., 2011; Seeleib-Kaiser et al., 2012). Employees in public administration and financial services usually have *high* educational levels and *general skills* that are not bound to specific firms (ISCO groups 1–3: managers, professionals, technicians and associate professionals). *General* and hence portable *skills* paired with *low* educational levels are dominant in personal services and the hospitality sector (ISCO groups 4, 5 and 9: clerical support workers, service and sales workers, elementary occupations). Manufacturing firms demand employees with *higher* firm- or industry-*specific skills* (ISCO group 8: plant and machine operators and assemblers) and employees with *lower* firm- or industry-*specific skills* (ISCO group 7: craft and related trade workers), while the majority of employees in the construction sector belong to the latter group. At the same time, one can distinguish between sectors with powerful trade unions and strong social partnerships (public sector and manufacturing) in contrast to those with weak trade unions and pluralistic social partnerships (personal services and hospitality). In terms of industrial relations, the Institutional Characteristics of Trade Unions, Wage Setting, State Intervention and Social Pacts (ICTWSS) database (Visser, 2013) and various Eurofound/European Industrial Relations Observatory (EIRO) reports include union density and collective bargaining coverage for different countries and sectors.

With regard to occupational pensions, I look at coverage rates and (employer) contributions. The article uses national resources because comparative data are not available. Nevertheless, the results are comparable to the extent that the article examines relative and not absolute coverage rates and contributions. Coverage rates show how many employees possess an occupational pension plan. Depending on data availability, contribution levels are presented in euros and/or in the percentage of wages. In the country studies, national averages are identified. For the sector studies, Tables 5–7 summarize the results of whether certain characteristics show values above (+), below (−) or similar (+/−) to the country averages. In the following, ‘high’ coverage rates and ‘high’ employer contributions only refer to the country averages. This does not imply ‘high’ in the sense that they satisfy the needs of employees and pensioners. Although contribution rates do not fully reflect the level of future benefits, they are a proxy for the generosity of occupational pension schemes for today’s employees.

## The institutional setting of pensions and the scope for occupational pensions

Before analysing occupational pensions, the overall pension system with its *pillars* (provider of the pension) and *tiers* (function of the pension; see Ebbinghaus, 2011a) clarifies the scope for and role of occupational pensions. The state takes responsibility for the first public pension pillar and mainly social partners or the employer for the occupational pension second pillar. The third individual pension pillar is not part of the analysis, as the take-up is left to the individual. Within each pillar, pensions can have three functions for old-age income security. They can guarantee a basic or means-tested income (first tier), maintain the living standard (second tier) usually via earnings-related state pensions or occupational pensions, and they can serve as a top up (third tier) in the form of additional personal savings.

Germany (still) represents a Bismarckian social insurance system with a dominant pay-as-you-go (PAYGO)-financed public pension system that includes the first and second tiers. Nevertheless, recent reforms have cut public benefits and led to a partial path departure towards the liberal model, making additional occupational pension savings necessary for status maintenance (Bridgen and Meyer, 2014; Ebbinghaus et al., 2011). Social partners succeeded in extending the role of occupational pensions during the reform processes of the 2000s, hoping for new power via collectively negotiated pensions and collective pension schemes (Wiß, 2011). Since 2001, employees have been entitled to request from their employers the conversion of parts of their salary into an occupational pension scheme, known as salary sacrifice (*Entgeltumwandlung*). A collective agreement determining the specific details is necessary when collectively negotiated wages are converted in this way (*Tarifvorbehalt*).

Italy, as another country with a Bismarckian pension system, differs from Germany in terms of higher public pension benefits, making additional occupational pensions for a decent old-age living standard almost superfluous for life-long, full time workers. As in Germany, the far-reaching pension reforms of recent decades have lowered public pension benefits. Thanks to the traditional mandatory and employer-only financed severance pay, *Trattamento di fine rapporto* (TFR),^3^ already existent employer contributions have been used since 2005 as an ‘institutional gate’ for the development of occupational pensions (Jessoula, 2011). TFR contributions are automatically converted into an occupational pension, mainly based on collective agreements, unless the employee insists on keeping the traditional TFR. Similar to Germany, trade unions associated with a boost of occupational pensions based on collective agreements hope for a democratization of capitalism and more power (Natali and Rhodes, 2008: 37).

The United Kingdom, with a mature multipillar pension system, comes with a comparatively low contributory basic state pension in combination with an earnings-related state second pension, replacing the State Earnings Related Pension Scheme from 1978 (Bridgen and Meyer, 2011: 265ff). It is possible to opt out of the state second pension and instead to take up an occupational pension. This system is currently changing and will be replaced with a flat-rate basic pension covering the first tier, typical for a liberal welfare regime, together with auto-enrolment in occupational pensions and the right to opt out. Although occupational pensions in Britain were voluntary, British trade unions did not force their development (Bridgen and Meyer, 2005: 772), except in the public sector.

Denmark, as a prime example of a multipillar pension system, offers a quite generous tax-financed basic public pension system with an additional prefunded flat-rate public pension (*Arbejdsmarkedets Tillægspension* (ATP)) partly covering the second tier. For status maintenance, occupational pensions organized sector-wide by collective agreements have been a very important part of old-age income since the 1980s and 1990s. Trade unions were an important driving force for the boost of occupational pensions, although they favoured at the beginning a central union-controlled pension fund over sector-wide collective agreements (Andersen, 2011).

## Occupational pensions within countries

### Germany: growing importance of voluntary occupational pensions

Owing to its voluntary character, occupational pension coverage in the private sector is lower (50%) compared with the public sector, where all employees other than civil servants are covered by compulsory occupational pensions based on a collective agreement (for details, see Wiß, 2011).^4^

In the private sector, the majority of occupational pension schemes are currently based on collective agreements, except for small firms and managers with individual working contracts. The benefits depend on contributions and investment returns (defined contributions (DC)) with a minimum guarantee (nominal value of contributions), whereas managers and senior clerks are joining more generous defined benefit (DB) schemes. In addition to higher coverage, public sector employees also profit from very high employer contributions, which make up four-fifths of overall contributions (7.86% of salary; Wiß, 2011). Table 1, based on a survey, provides an overview of the occupational pension coverage rates in different sectors. They are very high in the public sector thanks to the mandatory occupational pension scheme, in the financial sector due to employer-sponsored schemes with very high employer contributions and in manufacturing despite its voluntariness. The social partners in the German chemicals industry are well known as pioneers regarding occupational welfare, and superannuation funds have been a tradition lasting a century (Wiß, 2012a). Thanks to the above-average employer contributions in manufacturing, around 80 percent of employees in the chemicals industry and 75 percent in the metals industry are covered by an occupational pension (Gesamtmetall, 2008; Wiß, 2011: 224). By contrast, the very low coverage rates in the hospitality sector as well as in administrative and support services (23%) correspond with below-average employer contributions.

**Table 1.** Occupational pension coverage 2011 (% of employees) and annual employer contributions 2008, Germany.

	Coverage rate	Employer contribution per pension plan (euros)	Employer contribution (% of gross wages)
Public sector	100	2270	3.8
Finance and insurance	84	3430	8.1
Manufacturing	63	2339	3.7

Construction industry	43	988	1.0
Hospitality sector	26	577	0.6
Administrative and support services	23	1273	0.7

Sources: Coverage rate: TNS Infratest (2012); employer contribution: own calculations based on Statistisches Bundesamt (2011).

The average coverage rate (61%) consists of 12.2 million employees in the private and 5.2 million employees in the public sector (and 28.7 million employees covered by statutory social insurance). The average annual employer contribution per occupational pension plan amounts to €2124, and the average employer contribution is 3.3 percent of gross wages.

### Italy: low coverage rates but high employer contributions

Despite their initial scepticism, Italian trade unions later pushed for closed pension funds based on collective agreements in order to gain new organizational power via negotiations and participation in the management of occupational pensions (Natali and Rhodes, 2008). Similar to Germany, the newly introduced pension plans offer defined contribution benefits with a nominal value guarantee of contributions. Only employees in the financial sector receive more generous DB pensions (Jessoula, 2011: 170–171). In 2011, around 20 percent of employees in the private and public sectors had an occupational pension (COVIP, 2012). In detail, more employees are covered by occupational pensions in the energy (90%), chemicals (80%) and metals (44%) industries compared with employees in the construction industry (6%) and other services (8%; see Table 2). Surprisingly, coverage rates for employees in the public sector are in general rather low (below 5%; COVIP, 2012: 38), but above the average in former public areas such as the national railway (42%), municipal utilities (60%) and the Italian post (62%; COVIP, 2012: 212). As a proxy for coverage rates in the banking and insurance sector, I refer to pre-existing pension funds that are mainly used by banks and insurance firms. Although closed for new members since 2003, 88 percent of employees are covered (COVIP, 2012: 156).

**Table 2.** Occupational pension coverage (% of employees) and contributions to closed pension funds (% of salary), Italy 2011.

	Coverage rate	Employer contribution	Total contribution
FONDENERGIA (energy industry)	90	1.9–2.0	10.3–10.9
FONCHIM (chemicals industry)	80	1.3–1.7	9.4–10.1
FONDOPOSTE (Italian post)	62	1.5	8.9–9.4
COMETA (metals industry)	44	1.2–1.5	9.3–9.9

PREV.I.LOG (logistics enterprises)	9	1	8.9
ESPERO (schools/education)	8	–	–
FONTE (other services and tourism)	8	0.6–1.6	8.0–9.5
PREVEDI (construction industry)	6	1	8.9

Source: COVIP (2012: 212–213).

TFR: *trattamento di fine rapporto.*

The average coverage rate for all closed pension funds in 2011 is 37 percent, the average employer contribution 1.1–2.0 percent and the average total contribution (employer’s TFR contribution of 6.91% + additional employer and employee contributions) 9.1–10.3 percent (own calculation based on COVIP, 2012).

The average overall contribution to collective pension funds is 9.1–10.3 percent of gross salary consisting of 6.91 percent mandatory TFR employer contributions together with additional employer and employee contributions. Employees in the financial sector profit from generous contributions, amounting to €7000 annual contributions per employee including a high employer’s share (COVIP, 2012: 158). In comparison with other countries, the employer-sponsored TFR leads to very low additional contributions and therefore only to slight deviations across sectors. Nevertheless, evidence for lower employer and total contribution rates for occupational pensions in other services such as tourism and construction compared with manufacturing and finance and insurance exists (Table 2). Crucial for the high coverage rates in Italy are the collective agreements and collective pension funds negotiated by social partners that make use of TFR contributions (Jessoula, 2011: 168).

### United Kingdom: medium to high coverage rates despite low public pensions

The mature occupational pension system in the United Kingdom offers voluntary occupational pension plans, although it is currently changing to more compulsory versions, thanks to a recently introduced auto-enrolment mechanism. The voluntary character is one of the main reasons for only medium to high coverage rates (47% in 2012) despite very low public pensions. In the past, a coalition of employers, insurers and the Labour government successfully intervened for more generous public pensions instead of more regulations for supplementary pensions (Bridgen and Meyer, 2011: 273–274). After a change in government and in times of tight public budgets, the Conservative-Liberal government (2010–2015) aimed to make the basic state pension more sustainable, less complex and supported occupational pension savings.

The number of employees in the private sector covered by an occupational pension plan has been decreasing over the past 20 years, reaching 32 percent in 2012 in contrast to an increase in the public sector (83% in 2012; Office for National Statistics (ONS), 2012: chapter 3). The variety in coverage rates among economic sectors is similar to those in Germany and Italy. The highest coverage rates are in public administration and finance and insurance followed by manufacturing (Table 3). Administrative and support services and especially the hospitality sector show below-average coverage rates.

**Table 3.** Employer-provided pension scheme coverage (% of employees), United Kingdom 2012.

	Coverage rate
Public administration and defence	91.2
Finance and insurance	71.5
Manufacturing	48.6

Construction	29.4
Administrative and support services	13.7
Hospitality sector	5.1

Source: Own calculations based on ONS (2013b).

DB: defined benefit; DC: defined contributions.

The average coverage rate for public and private employees is 46.5 percent. Coverage rates include all employer-provided pensions (DB and DC schemes, group personal pensions and group stakeholder pensions).

**Figure 1.**
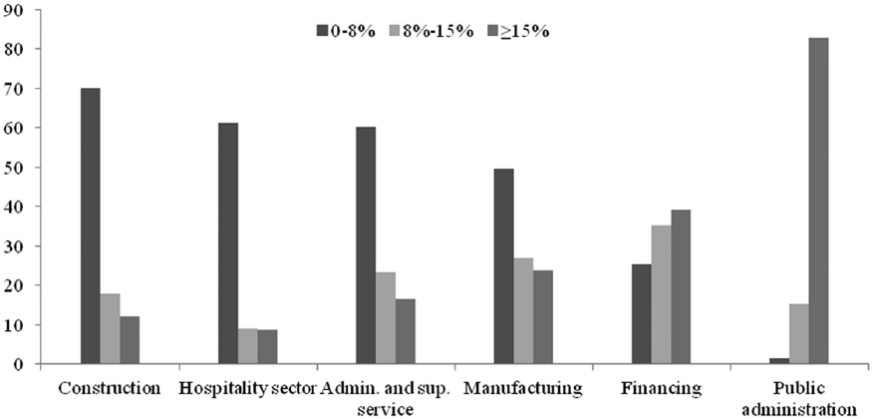
Employer contributions to workplace pensions (% of gross salary), United Kingdom 2012. Source: Own calculations based on ONS (2013b).

The quality of pension plans also systematically differs across sectors. In terms of contributions, employers sponsor the most in the public sector and in finance and insurance, where 83 and 39 percent of employees, respectively, receive employer contributions higher than 14 percent (Figure 1). The lowest contributions are paid by employers in construction, hospitality and administrative and support services. Separated by benefits, private sector employers contribute on average 6.6 percent to DC schemes and 14.2 percent to DB schemes, which dominate in the public sector and in manufacturing (ONS, 2013a).^5^

### Denmark: well-established occupational pension schemes due to quasi-mandatory collective agreements

In contrast to Germany, Italy and the United Kingdom, occupational pensions in Denmark are quasi-mandatory with near-universal coverage (⩾90%) based on sector-wide collective agreements (Andersen, 2011), thus resulting in negligible sectoral differences. Prior to the equally distributed occupational pensions across all sectors, employees in the manufacturing industry and in banks joined the first two sector-wide pension funds in 1900 and 1912, respectively (see Andersen, 2011: 192). After the Second World War, nationwide collectively negotiated occupational pension schemes were established for employees in the public sector (1946) and for engineers (1953). The non-introduction of a public pension scheme fully covering the second tier left space for voluntary sector-wide occupational pensions (crowding-in). With the public sector ahead, in 1986, 48 percent of public sector employees had an occupational pension plan compared with 34 percent in the private sector (Andersen, 2011: 193). In the 1980s, the government initiated the expansion of occupational pensions via collective agreements, while the trade unions welcomed the expansion of funded pensions. As a result, collective agreements were negotiated at the beginning of the 1990s in order to expand occupational pensions to all employees not yet covered (Andersen, 2011: 193–194).

Today, in contrast to coverage, some variation exists in terms of contribution level. In the public sector, occupational pensions are employer-only financed with a contribution rate of 12 percent (Organisation for Economic Co-operation and Development (OECD), 2009: 182), whereas they range between 9 and 15 percent in the private sector, of which employers usually contribute two-thirds (OECD, 2009). Total contributions are, similar to the other studied countries, above average in the financial sector and manufacturing and below average in other services and the construction industry (Table 4). The universal flat-rate basic state pension explains some of the cross-sectoral differences in occupational pensions, where replacement rates from the public system are higher for low-wage earners in the construction industry and hospitality sector. Consequently, lower occupational pension contributions are observed compared with low public replacement rates for high-income earners in the financial sector, resulting in higher occupational pension contributions.

**Table 4.** Average annual contributions to occupational pensions (Danish kroner), Denmark 2010.

	Total contributions	Contribution (% of income)^a^
Finance and insurance	78,536 (~€10,500)	15.7
Industrial sector	45,672 (~€6100)	12.4
		
Public administration, education and health	38,796 (~€5200)	12.1
		
Culture and other services	37,889 (~€5100)	11.8
Trading and transport	37,951 (~€5100)	11.2
Construction industry	35,395 (~€4800)	11.8

Source: Forsikring & Pension (2012).

The average annual total contribution to occupational pensions in all sectors is 39,425 Danish krone (~€5300; own calculations based on Forsikring & Pension, 2012).

a Total contributions to occupational and private pensions as a % of gross salary with a median of 11.8 percent.

To sum up, in all our countries except Denmark, occupational pensions largely differ across sectors. Owing to very strong trade unions negotiating industry-wide collective agreements, occupational pensions are more equally distributed in Denmark. To show evidence for our theoretical considerations, the next section illustrates the factors influencing occupational pensions, both economic individual power and political collective power, in similar sectors across countries.

## Occupational pension similarities across countries

### Public sector

In the public sector, we find very high coverage rates in all countries together with high employer contributions, except for Italy (see Table 5). Although detailed data for coverage rates across sectors are missing for Denmark, equal coverage rates for all sectors (indicated with +/−) are assumed, referring to near-universal coverage rates and equally distributed pension plans in the literature (Andersen, 2011; OECD, 2009; Trampusch, 2013). Confirming the skill argument, employees with high-general skills dominate (>60%) in the three countries with widespread and generous occupational pensions. The exceptionally low levels of coverage and employer contributions in Italy are related to a high share of employees with low-general skills, representing half of the public administration’s workforce. Nevertheless, in former public areas in Italy with very strong unions such as railways, public transport and the Italian post, occupational pensions are clearly above the country average (COVIP, 2012: 212). In addition to high-general skills, very strong trade unions compared with the private sector are associated with high occupational coverage rates and contributions in Germany, the United Kingdom and Denmark. Despite the high share of employees with low-general skills in the United Kingdom (36%), coverage rates in the highly unionized public administration are above the country average. In Denmark, the collective power plays a more important role than skills. Almost one-third of employees have only low-general skills, but basically all public employees are covered (Trampusch, 2013: 44) and contributions are fully paid by employers for all employees, including the less qualified.

**Table 5.** Occupational pensions, industrial relations and skills in the public sector and in finance and insurance.

	Public sector				Finance and insurance			
	DE	IT	UK	DK	DE	IT	UK	DK
*Occupational pensions*								
Coverage rate	+	−	+	+/−	+	+	+	+/−
Employer contributions	+	−	+	+	+	+	+	+
*Industrial relations*								
Union density	+	+	+	+	−	−	−	+/−
Collective bargaining coverage	+	+	+	+	+	+	+/−	+
*Skills*								
High-general	64 (+)	31 (−)	61 (+)	62 (+)	38 (−)	69 (+)	69 (+)	83 (+)
High-specific	1 (−)	1 (−)	1 (−)	0 (−)	0 (−)	0 (−)	0 (−)	0 (−)
Low-specific	2 (−)	1 (−)	2 (−)	n (−)	0 (−)	0 (−)	0 (−)	0 (−)
Low-general	24 (−)	49 (+)	36 (+/−)	31 (−)	62 (+)	32 (−)	31 (−)	16 (−)

Sources: Occupational pensions: see section ‘Occupational pensions within countries’; industrial relations: EIRO (2011a, 2011b, 2012b) and Visser (2013); collective bargaining coverage Germany: Ellguth and Kohaut (2013), UK: Department for Business Innovation and Skills (BIS) and Office for National Statistics (ONS) (2013); skills: own calculations based on the EUROSTAT LFS.

+ : above country average; −: below country average; +/−: country average (if the value is within ±2% of the country average for skills); n: no information; DE: Germany; IT: Italy; UK: United Kingdom; DK: Denmark; LFS: Labour Force Survey.

In terms of skills, sums below 100 are due to missing data.

### Finance and insurance

In finance and insurance, coverage rates and employer contributions are generous in all countries. Employees with high-general skills in Italy, the United Kingdom and Denmark (representing 69%–83% of all employees) are in powerful positions to ask for employer benefits such as occupational pensions. With high contributions, employers aim to attract and retain highly qualified employees. Moreover, prefunded financial products such as occupational pension plans are in the interest of financial institutions. Table 5 shows above-average levels of coverage and contribution rates and only low levels of union power. At first glance, the below-average union density of only 15 percent in Germany (2011) and 17 percent in Italy (1997; Wiß, 2012a: 169; Visser, 2013) hardly explains the very well-established occupational pension schemes in banks and insurance firms. However, if we refer to the private sector averages, density rates as well as bargaining coverage rates are above average, indicating relatively well-established industrial relations systems. Only in Germany do the majority of employees in the finance sector have low-general skills (62%). Their above-average coverage rate seems to be related to widespread collective agreements (collective bargaining coverage rates are 79% in West and 64% East Germany (Ellguth and Kohaut, 2013)), which also comprise occupational pensions (Wiß, 2011).

### Manufacturing industry

In the manufacturing industry, the share of employees with high- and low-specific skills is above the national averages in all countries, accounting for 41–57 percent of the sector’s workforce (see Table 6). In order to motivate employees to invest in these specific skills, despite their risky nature and non-portability, employers voluntarily offer occupational pension plans for the majority of their economically strong workforce. In terms of collective power, union density rates for manual workers in Germany (30% in 2004) are twice the number for non-manual workers (14% in 2004; Visser, 2013). The chemical and metal industries in Italy with strong unions show above-average occupational pension coverage rates (Jessoula, 2011: 168). Only in the United Kingdom is union power in manufacturing below the country average union power, but it is still higher than the average union power in the private sector. Furthermore, although the relative share of specifically qualified employees in manufacturing (41%) is above the country average in the United Kingdom, employees with high-general skills are the biggest group in manufacturing in absolute terms (44%). In sum, in manufacturing, there is a strong association of individual economic power of specifically qualified employees and trade unions’ collective power with widespread occupational pension plans and medium to high employer contributions.

**Table 6.** Occupational pensions, industrial relations and skills in manufacturing and construction.

	Manufacturing				Construction			
	DE	IT	UK	DK	DE	IT	UK	DK
*Occupational pensions*								
Coverage rate	+	+	+	+/−	+/−	−	−	+/−
Employer contributions	+	+/−	+/−	+	−	−	−	−
*Industrial relations*								
Union density	+	+	−	+	+	+	−	+
Collective bargaining coverage	+/−	+	−	+	+	n	−	n
*Skills*								
High-general	36 (−)	27 (−)	44 (−)	39 (−)	28 (−)	15 (−)	32 (−)	19 (−)
High-specific	14 (+)	20 (+)	16 (+)	20 (+)	6 (+/−)	6 (+/−)	3 (+/−)	6 (+/−)
Low-specific	32 (+)	37 (+)	25 (+)	23 (+)	55 (+)	71 (+)	50 (+)	64 (+)
Low-general	21 (−)	17 (−)	18 (−)	20 (−)	14 (−)	10 (−)	17 (−)	14 (−)

Sources: Occupational pensions: see section ‘Occupational pensions within countries’; industrial relations: Clarke et al. (2005), EIRO (2010) and Visser (2013); collective bargaining coverage Germany: Ellguth and Kohaut (2013), UK: BIS and ONS (2013); skills: LFS.

+ : above country average; −: below country average; +/−: country average (if the value is within ±2% of the country average for skills); n: no information; DE: Germany; IT: Italy; UK: United Kingdom; DK: Denmark; LFS: Labour Force Survey.

In terms of skills, sums below 100 are due to missing data.

### Construction industry

In the construction industry, the level of employer contributions is below the country averages (see Table 6) and coverage rates are below average in Italy and the United Kingdom. A closer look at the two groups of specific skills shows that in contrast to manufacturing, only employees with low-specific skills are above the country averages. They amount to 55 percent in Germany, 71 percent in Italy, 50 percent in the United Kingdom and 64 percent in Denmark and by far exceed the country averages (all below 15%). This finding confirms the low skill level in the construction industry observed by Mares (2003b: 249–50). However, why are coverage rates medium to high in Germany and Denmark despite many employees with low-specific skills? As in the other sectors in Denmark, strong trade unions have been able to negotiate industry-wide collective agreements for construction workers, but with lower employer contributions. In Germany, all workers in West Germany are covered by mandatory occupational pensions based on extended collective agreements, thanks to powerful trade unions (Wiß, 2011). Table 6 only shows an average coverage rate because this pension scheme does not apply to workers in East Germany. Italy remains an outsider with low coverage rates despite strong unions. Although fewer than 10 percent of employees have an occupational pension plan, traditional welfare funds established via a national collective agreement provide to all construction workers occupational welfare such as a Christmas bonus, holiday pay and additional pension contributions depending on the length of service.^6^

### Hospitality and other services

Although the data on occupational pensions in Italy for the hospitality sector are meagre, as a proxy the article refers to the coverage rate and contributions of the pension fund FONTE (see Table 2) that covers, among others, employees in tourism. Furthermore, we do not expect contrasting results compared with other countries and other services such as administrative and support services (e.g. cleaners and security guards) due to weak trade unions and low skilled employees. In both sectors and all countries except Denmark only scattered and rudimentary occupational pension schemes with low employer contributions are apparent (see Table 7). Since 58–89 percent of all employees across countries have low-general skills, their lack of individual economic power places them in a poor position to negotiate occupational benefits. Furthermore, employers only have a low interest to invest in occupational pensions for employees with low portable skills. The powerless trade unions in the hospitality sector (union density of 5% in Germany and 4% in the United Kingdom (Pohl, 2006; Visser, 2013)) and in administrative and support services (e.g. union density of 11% in the United Kingdom in 2012 (BIS and ONS, 2013)) are not able to compensate for the lack of individual economic power and negotiate additional occupational benefits on a par with employers. Although trade unions seem to have only moderate power, the positive case of Denmark, with comparatively high coverage rates, is related to medium to strong unions in the service sector and business services (65%–67%; Visser, 2013) and medium to high collective bargaining rates roughly mirroring the private sector average of 77 percent (EIRO, 2012a, 2012d). Danish collective agreements regulating occupational pensions covering three-quarters of the workforce, even in sectors with high shares of small enterprises, mitigate the usually negative impact of small firms on occupational welfare.

**Table 7.** Occupational pensions, industrial relations and skills in the hospitality sector and administrative and support services.

	Hospitality sector				Administrative and support services			
	DE	IT	UK	DK	DE	IT	UK	DK
*Occupational pensions*								
Coverage rate	−	−	−	+/−	−	−	−	+/−
Employer contributions	−	−	−	−	−	−	−	−
*Industrial relations*								
Union density	−	−	−	−	−	−	−	+/−
Collective bargaining coverage	−	+^a^	−	+/−	+/−^a^	+^a^	−	+/−
*Skills*								
High-general	20 (−)	18 (−)	35 (−)	10 (−)	25 (−)	13 (−)	32 (−)	27 (−)
High-specific	01 (−)	00 (−)	01 (−)	00 (−)	05 (+/−)	02 (−)	03 (+/−)	00 (−)
Low-specific	02 (−)	03 (−)	01 (−)	00 (−)	08 (−)	04 (−)	03 (−)	04 (−)
Low-general	80 (+)	80 (+)	66 (+)	89 (+)	58 (+)	77 (+)	58 (+)	61 (+)

Sources: Occupational pensions: see section ‘Occupational pensions within countries’; industrial relations: EIRO (2012a, 2012c, 2012d) and Visser (2013); collective bargaining coverage Germany: Ellguth and Kohaut (2013), UK: BIS and ONS (2013); skills: LFS.

+ : above country average; −: below country average; +/−: country average (if the value is within ±2% of the country average for skills); n: no information; DE: Germany; IT: Italy; UK: United Kingdom; DK: Denmark; LFS: Labour Force Survey.

a : high collective bargaining coverage rates in Germany and Italy result from extended collective agreements containing only minimum wages. In terms of skills, sums below 100 are due to missing data.

To summarize, the sector-specific case studies of this section demonstrated that economic individual power and/or political collective power are associated with widespread and generous occupational pension schemes. In contrast, if employees possess neither economic individual power nor political collective power, coverage rates and contributions are low.

## Conclusion

The analysis in this article provides a detailed comparison of occupational pension systems and the situation of different employee groups in the selected countries. Although national pension systems as well as occupational pension schemes differ across countries in terms of their institutional set-ups, coverage rates and contributions, significant similarities in the same economic sectors across countries were found when using the national averages as benchmarks. Germany, Italy and the United Kingdom show similar patterns of cross-sectoral differences despite highly diverse public/private mixes. Coverage rates and employer contributions are high in the public sector, finance and insurance and manufacturing in these countries. Conversely, employees are only rarely covered by occupational pensions, and if so, only with low employer contributions in the construction industry, the hospitality sector and administrative and support services. By contrast, Denmark shows a more homogeneous national occupational pension model, thanks to its quasi-mandatory collective agreements in all economic sectors.

The article shows that neither skills nor political power resources of employees alone is sufficient to explain the variation in occupational pensions across countries and sectors. Additionally, the arguments are applied to sectoral comparisons instead of country comparisons. Where research has thus far mainly treated both strands as rivals, their shortcomings are eliminated with their complementarity. The article demonstrates that individual skills materialize in economic power of employees and thereby expands traditional power resources theories, which mainly consider political collective power. As a result, two paths for widespread occupational pension schemes with above-average employer contributions are apparent. First, owing to their educational status, employees with high-general and high-specific skills can transfer their human capital into economic individual power, thereby negotiating generous occupational pensions with their employers which are in need of these skills (e.g. in finance and insurance and manufacturing). Second, political collective power interventions, depending on the power of trade unions, can equip economically weak employees with occupational pensions despite their low-general skills. This is the case for the construction industry, hospitality sector and administrative and support services in Denmark, the financial sector and construction industry in Germany and partially for the public sector in the United Kingdom. Furthermore, the sector-specific case studies illustrate that economic individual power and political collective power are very weak in the construction industry, hospitality sector and administrative and support services resulting in scattered and rudimentary occupational pensions.

For future prospects, national models and country differences still matter for public pensions and the importance of occupational pensions. Nevertheless, this article highlights within-country differences in order to show inequalities across sectors, often hidden in aggregated country-level analyses. It is likely that the growing importance of occupational welfare is intensifying processes of dualization, depending on the economic sector in which employees are working, unless trade unions negotiate sector- or nationwide collective occupational pension schemes or political actions declare them mandatory.

## Notes

1. Seeleib-Kaiser and Fleckenstein (2009) conclude that managers are protagonists and not consenters in occupational family policy. Their result is not surprising since it is based on (1) only stock-listed companies and (2) a survey of managers who barely would admit the introduction of image-supporting occupational family benefits due to works councils or union pressure.

2. But see the country chapters in Ebbinghaus (2011b) for partial departures from the pension regimes especially for Germany and the United Kingdom.

3. The *Trattamento di fine rapporto* (TFR) is an obligatory severance pay, financed by the employer and paid to all employees when they retire or leave the company (for details, see Jessoula, 2011).

4. Civil servants enjoy a tax-financed pension with a maximum replacement rate of 72 percent (until 2003: 75%) after 40 years of employment (Maurer et al., 2009). By contrast, public sector employees are insured with the normal contribution-based statutory pension insurance providing lower benefits. As compensation for this disadvantage compared with civil servants, public employees are covered by mandatory occupational pensions (Ebbinghaus et al., 2011).

5. Employer contributions are higher for defined benefit (DB) schemes (Office for National Statistics (ONS), 2013a) of which many are closed for new members (Bridgen and Meyer, 2011). Therefore, new occupational plan members are mainly enrolled in defined contributions (DC) schemes with lower employer contributions, but this does not change the unequal level of employer contributions across economic sectors.

6. For further information about paritarian social funds offering occupational welfare for the construction industry in Europe, see http://www.paritarian-funds-construction.eu.
